# Correction to “Upregulation of HLA‐II Related to LAG‐3^+^ CD4 ^+^ T Cell Infiltration is Associated With Patient Outcome in Human Glioblastoma”

**DOI:** 10.1111/cas.70135

**Published:** 2025-07-01

Guo W, Peng D, Liao Y, et al. Upregulation of HLA‐II Related to LAG‐3^+^CD4^+^ T cell Infiltration is Associated With Patient Outcome in Human Glioblastoma. *Cancer Science* 2024;115:1388–1404. https://doi.org/10.1111/cas.16128.

In the above article, Figure 1c is incorrect. The correct image is shown below:

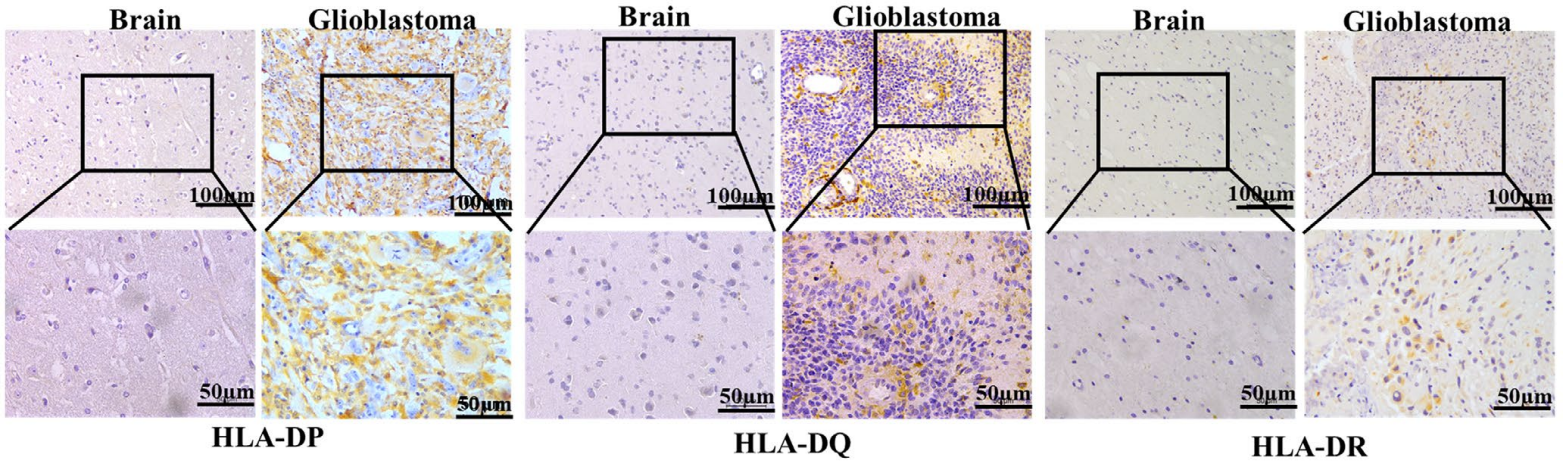

We apologize for this error.

